# Optic nerve glioma successfully treated with definitive intensity-modulated radiotherapy in a limited-resource setting: a case report

**DOI:** 10.3332/ecancer.2024.1800

**Published:** 2024-11-14

**Authors:** Judith Naa Odey Tackie, Ernestina Schandorf, Patrick Bankah, Hafisatu Gbadamosi, Joseph Daniels, Mary Ann Dadzie

**Affiliations:** 1 National Centre for Radiotherapy, Oncology and Nuclear Medicine, Korle Bu Teaching Hospital, Accra, Ghana; 2 Department of Child Health, Korle Bu Teaching Hospital, Accra, Ghana; 3 Department of Neurosurgery, Korle Bu Teaching Hospital, Accra, Ghana; 4 Department of Radiology, Korle Bu Teaching Hospital, Accra, Ghana; a https://orcid.org/0000-0002-1466-150X

**Keywords:** optic nerve glioma, radiotherapy, radiotherapy, intensity-modulated, central nervous system tumours, pilocytic astrocytoma, neurofibromatosis 1, pediatric radiation oncology, vision disorders

## Abstract

Optic nerve gliomas (ONG) are benign central nervous system tumours and the most common tumours of the optic nerve in children, often occurring before age 20. These tumours are slow-growing and can be treated with surgery and/or radiation therapy. Surgical resection is, however, associated with significant morbidity and loss of vision in the affected eye. The successful management of ONG requires a multidisciplinary, individualised approach aimed at preserving vision. This case report discusses an 11-year-old Ghanaian male diagnosed with left ONG in July 2023. He presented with blurred vision in the left eye, mild proptosis and poor vision, but no pain, headaches or neurological deficits. A contrast-enhanced magnetic resonance imaging of the orbit showed diffuse fusiform enlargement of the left optic nerve (35 × 12 × 11 mm). The successful management of ONG, in this case, using intensity-modulated radiotherapy demonstrates the potential of advanced radiotherapy techniques in preserving vision and achieving tumour control in pediatric patients, even in resource-limited settings. Continuous follow-up and a multidisciplinary approach are essential for managing the long-term outcomes and ensuring the best possible quality of life for these patients.

## Introduction

Optic nerve gliomas (ONG) are benign central nervous system (CNS) tumours, accounting for approximately 1%–5% of all gliomas and are the most frequent tumours of the optic nerve [1], often associated with neurofibromatosis type 1 (NF1) [2]. There are two types of ONG: juvenile benign pilocytic astrocytoma and malignant glioblastoma of adulthood. These tumours commonly occur in children and females under the age of 20 years, and mostly in patients below the age of 10 years. Among patients with ONG, 20%–50% are associated with NF-1 and these cases are more likely to have lesions involving unilateral or bilateral optic nerves [3, 4]. Sporadic ONG commonly involve the chiasma or hypothalamus. Studies have shown 42% of gliomas involve the optic nerves and chiasm, 33% the hypothalamus and chiasm and 25% the chiasm alone [4]. Those associated with NF1 have neurofibromin, a tumour suppressor gene on chromosome 17q that is inactivated, turning on rat sarcoma (RAS) signaling pathways [5]. Generally, ONG have a better prognosis than gliomas involving the optic chiasm. Also, gliomas of the anterior chiasm are associated with better outcomes than those involving the posterior chiasm [1, 4]. The management of ONG is complex and multifaceted, requiring a highly individualised approach to balance the goals of tumour control and vision preservation.

## Case presentation

We present the case of an 11-year-old male child who was first seen at the National Centre for Radiotherapy, Oncology and Nuclear medicine, Korle Bu Teaching Hospital on 3 July 2023 with a diagnosis of left ONG. He was apparently well until his mother noticed protrusion of his left eye 3 months prior to presentation. There was no associated pain or headaches; however, he had blurred vision in the left eye. There was no associated strabismus, focal neurological motor deficits or symptoms of raised intracranial pressure. The patient had no significant past medical, surgical or family history. Birth history was unremarkable, and he achieved his developmental milestones at appropriate ages. Clinical examination revealed a well-looking, active child with a performance status of ECOG 1. He had mild proptosis of the left eye. There was impairment of the third cranial nerve (oculomotor nerve) of the ipsilateral side. His visual test showed VL-2/6 and VR-6/6. The patient had no evidence of stigmata of NF1.

**Figure 1.**
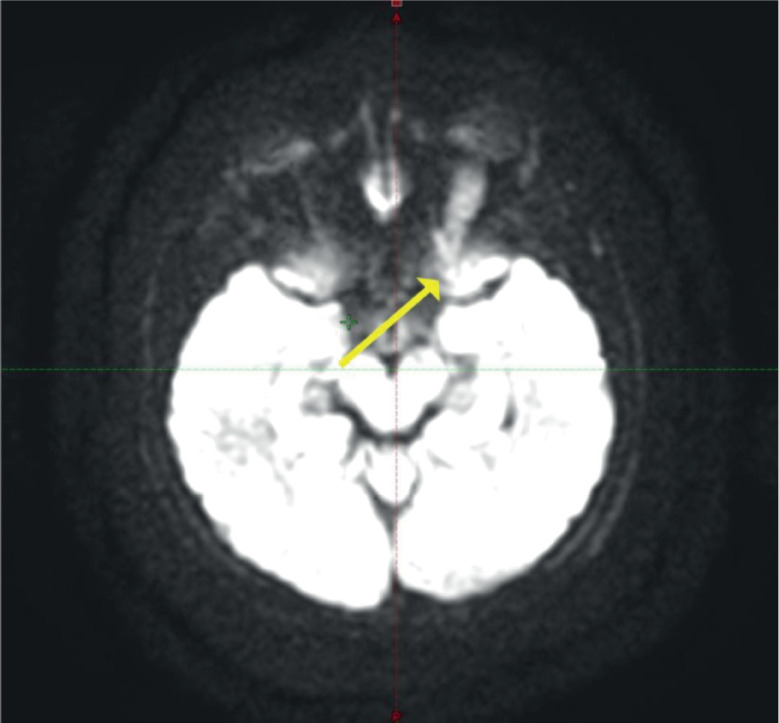
MRI of the brain and orbit showing an enlarged left optic nerve (yellow arrow).

Contrast-enhanced magnetic resonance imaging (C-EMRI) of the brain and orbit done on 5 June 2023 showed diffuse fusiform enlargement of the left optic nerve measuring (35 × 12 × 11) mm as illustrated in Figure 1. There was involvement of the entire intra-orbital segments of the left optic nerve. The lesion was T1 hypointense and T2 hyperintense. Left proptosis was noted with no extension to the optic chiasm or contralateral optic nerve. The brain parenchyma was unremarkable.

**Figure 2.**
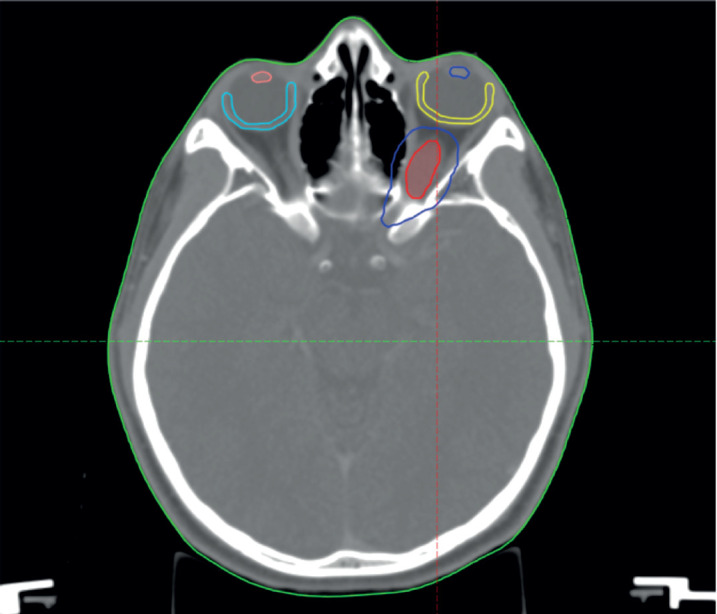
Diagram showing the GTV = red, PTV = blue, left macula = yellow, right macula = light blue, left lens = blue and right lens = orange.

**Figure 3.**
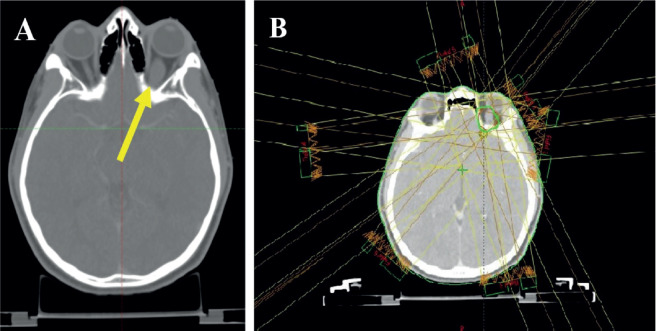
*Planning* CT *scan images of the patient. (a): Arrow shows hypodense fusiform enlargement of the left optic nerve.* (b): Contours and isodose coverage of the PTV and beam arrangements.

**Figure 4.**
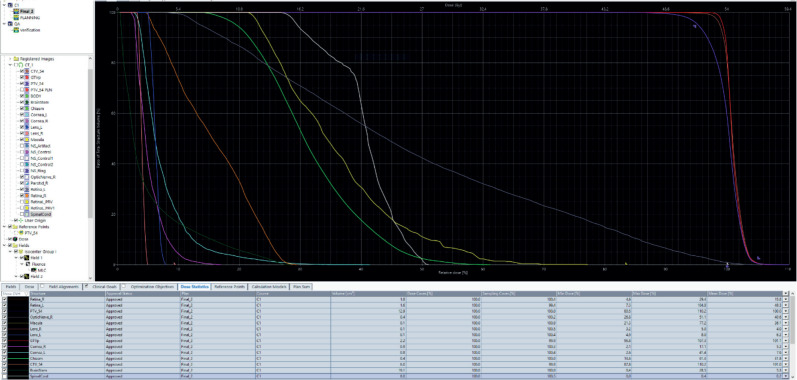
Diagram showing the dose volume histogram (DVH). Gross primary tumour volume (GTVp): red, PTV: purple, clinical target volume (CTV): brown, left retina: grey, right retina: orange, right optic nerve: white, left macula: yellow, right cornea: violet, left cornea: cyan, optic chiasm: bright green, left lens: blue, right lens: pink and brainstem: dark green.

The patient was discussed at a pediatric multidisciplinary tumour board resulting in a recommendation for the patient to be treated with definitive radiotherapy. A computed tomography (CT) scan was done in planning position with the child supine using a headrest and thermoplastic mask for immobilization. The magnetic resonance imaging (MRI) was fused with the planning CT scan to facilitate accurate target volume delineation. The gross tumour volume (GTV) was contoured, and planning target volume (PTV) margin generated with 5 mm outward expansion from the GTV (Figure 2). The prescribed dose to the PTV was 54 Gy in 27 fractions delivered via intensity-modulated radiotherapy (Figure 3). The maximum radiation doses to ipsilateral critical structures such as the left retina, macula, cornea and lens were 52, 43, 46 and 10 Gy, respectively (Figure 4).

The patient and his parents were counseled on the potential late toxicity of radiotherapy. He completed radiation treatment on 6 September 2023 without any acute toxicity. A C-EMRI done 3 months post radiation therapy (RT) showed reduction in the size of the diffuse left optic nerve lesion. Virtual assessment done also showed improvement in vision in his left eye. The patient is currently under post-treatment surveillance and receiving optimal survivorship care.

## Discussion

The natural history of ONG is usually indolent for NF-1-associated tumours and aggressive in spontaneous gliomas. These tumours may bridge through the optic foramen, with the nerve being infiltrated by tumour extending from the chiasm, walls of the third ventricle or hypothalamus.

### Clinical presentation and diagnosis

Clinical presentation of ONG includes worsening proptosis, strabismus, nystagmus, visual field impairment, acute loss of vison, ataxia, precocious puberty and developmental delay. Lesions involving the chiasm may extend to the hypothalamus causing diencephalic syndrome which is associated with emaciation. Patients with extension to the optic chiasm and beyond may present with symptoms of increased intracranial pressure (ICP), bitemporal field defects and endocrine dysfunction. Precocious puberty is commonly seen in tumours associated with NF1. Some patients may, however, be asymptomatic [6, 7].

In this case, an 11-year-old male presented with blurred vision in the left eye, mild proptosis and poor vision in the ipsilateral eye, without associated pain, headaches or other neurological deficits. The absence of systemic symptoms or NF1 stigmata is noteworthy as it can influence the treatment approach. Fundoscopy reveals optic disc swelling due to venous engorgement or pallor of the disc due to atrophy from compressive effects of the tumour. Chronic compression of the central retinal vein can lead to central retinal vein occlusion, resulting in venous stasis retinopathy and rubeosis iridis with neovascular glaucoma. Neurological examination may reveal partial or total vision loss or signs of ICP [1–4, 8].

Radiological findings on CT comprise an iso-dense fusiform enlargement of the optic nerve with erosion of the optic canal, less commonly a discrete mass arising from the optic nerve [9, 10]. Calcifications are rare compared to optic nerve meningiomas in which calcifications are seen in 20%–50% of cases [11]. The diagnosis of this patient was confirmed with C-EMRI, which revealed a diffuse fusiform enlargement of the left optic nerve, consistent with typical radiographic features of ONG. MRI is the imaging modality of choice for assessing the extent of disease and its extension to the optic chiasm and beyond. The course of the optic nerve is best evaluated on T1-weighted images. The tumour is iso-dense or hypo-dense on T1-weighted images and hyper-dense on T2-weighted images, enhanced on gadolinium contrast studies [7].

### Histopathology

Histopathological evaluation of most ONG reveals low-grade tumours, typically pilocytic or fibrillary astrocytoma. They most commonly range from piloid and stellate astrocytes with or without oligodendroglia to malignant astrocytoma to glioblastoma multiforme, which is rare. There is a rare subset of ONG that are more aggressive, presenting as pilomyxoid astrocytoma [12].

### Treatment approach

The treatment of ONG is tailored to the patient’s symptoms and the tumour’s progression. Given that ONG are generally slow-growing and benign, the main goal is to preserve vision and minimise treatment-related morbidity. In cases where vision is severely affected or the tumour is progressive, active intervention is warranted [6]. Management of ONG requires a multidisciplinary approach involving ophthalmologists, pediatric oncologists, neurosurgeons, oculoplastic surgeons and radiation oncologists. Treatment should be individualised based on symptoms, tumour extent and progression, NF1 status and patient preference.

Surgical resection of ONG may help reduce proptosis and pain but carries a substantial risk of vision loss [1]. Therefore, surgical intervention has typically been limited to obtaining a biopsy for diagnostic purposes or in case of significant mass effect; for relieving pressure on the optic nerve, chiasm or surrounding structures [12].

RT is generally deferred in children due to the risk of long-term radiation induced toxicity. However, RT remains an important definitive treatment option for well-selected sporadic ONG in older children, providing better local control and preserving functional vision. Studies have shown that stabilization of vision and limited disease progression can be achieved with RT. Techniques such as 3-D conformal radiotherapy, intensity-modulated radiotherapy (IMRT), stereotactic radiosurgery or proton therapy offer better local control and reduced late normal tissue toxicity, including secondary malignancy and radiation-induced moyamoya syndrome. Highly conformal RT to doses of 50.4 to 54 Gy has been shown to provide excellent local control [4, 13].

Upfront chemotherapy is the preferred first-line treatment for children with ONG, but it is associated with a risk of functional vision loss due to progression, requiring salvage treatment. Children who develop functional vision loss following chemotherapy and subsequently receive RT rarely recover functional vision [14]. The randomised Children’s Oncology Group A9952 study showed a 5-year progression-free survival (PFS) of 35% using carboplatin with vincristine and 48% using thioguanine, procarbazine, lomustine and vincristine in children with newly diagnosed progressive low-grade glioma. Cisplatin-based regimens have shown responses between 50% and 60%, with a 5-year PFS of 50% [4, 13].

### Outcome and follow-up

Generally, the prognosis of ONG is good with a better visual prognosis and reported 5-year overall survival rates between 70% and 90%. Even though the long-term morbidity is high, patients younger than 5 years at presentation with NF-1 have a better PFS rate [4, 15]. The patient in this case report showed no evidence of local relapse 11 months post-radiotherapy, underscoring the efficacy of IMRT in achieving local control. Long-term follow-up is crucial in pediatric patients to monitor for potential late effects of radiation, such as secondary malignancies or endocrinopathies and to ensure ongoing tumour control [16].

Given that IMRT allows for precise dose delivery and minimises radiation exposure to surrounding healthy tissues, the risk of acute and long-term toxicity is lower compared to conventional radiotherapy. However, the potential long-term toxicities that will be considered for follow-up in this patient include radiation-induced optic neuropathy and retinopathy which could result in loss of vision in the ipsilateral eye. Patient monitoring for the long-term outcome will focus on tumour control, visual function and quality of life. Long-term surveillance through imaging with MRI scans will be crucial to ensure that there is no progression or recurrence. Regular ophthalmological evaluations will be conducted to track changes in vision over time.

### Implications for resource-limited settings

This case highlights the feasibility and success of using advanced radiotherapy techniques like IMRT in a resource-limited setting for the management of ONG. It also underscores the importance of a multidisciplinary approach and the need for resource optimization to achieve optimal outcomes in managing complex CNS tumours in low-resource environments. Furthermore, it emphasises the potential for telemedicine and international collaborations to support local healthcare providers with expert consultations and treatment planning.

## Conclusion

The successful management of ONG in this case through IMRT demonstrates the potential of advanced radiotherapy techniques in preserving vision and achieving tumour control in pediatric patients, even in resource-limited settings. Continuous follow-up and a multidisciplinary approach are essential for managing the long-term outcomes and ensuring the best possible quality of life for patients with ONG.

## Conflicts of interest

The authors declare that they have no conflicts of interest.

## Funding

The authors declare that no funds, grants, awards or other support were received either during the conduct of the study or during the preparation of the manuscript.
